# The Many Faces of Kefir Fermented Dairy Products: Quality Characteristics, Flavour Chemistry, Nutritional Value, Health Benefits, and Safety

**DOI:** 10.3390/nu12020346

**Published:** 2020-01-28

**Authors:** Mohamed A. Farag, Suzan A. Jomaa, Aida Abd El-Wahed, Hesham R. El-Seedi

**Affiliations:** 1 Pharmacognosy Department, College of Pharmacy, Cairo University, Kasr El Aini St., P.B., Cairo 11562, Egypt; 2 Chemistry Department, School of Sciences & Engineering, The American University in Cairo, New Cairo 11835, Egypt; suzyaj@aucegypt.edu; 3 Department of Bee Research, Plant Protection Research Institute, Agricultural Research Centre, Giza 12627, Egypt; aidaabd.elwahed@arc.sci.eg; 4 Pharmacognosy Group, Department of Medicinal Chemistry, Uppsala University, Biomedical Centre, Box 574, SE-751 23 Uppsala, Sweden; 5 Al-Rayan Research and Innovation Center, Al-Rayan Colleges, Medina 42541, Saudi Arabia; 6 International Research Center for Food Nutrition and Safety, Jiangsu University, Zhenjiang 212013, China; 7 Department of Molecular Biosciences, The Wenner-Gren Institute, Stockholm University, SE 106 91 Stockholm, Sweden

**Keywords:** kefir, composition, physicochemical properties, sensory characters, nutritional value, biological effects

## Abstract

Kefir is a dairy product that can be prepared from different milk types, such as goat, buffalo, sheep, camel, or cow via microbial fermentation (inoculating milk with kefir grains). As such, kefir contains various bacteria and yeasts which influence its chemical and sensory characteristics. A mixture of two kinds of milk promotes kefir sensory and rheological properties aside from improving its nutritional value. Additives such as inulin can also enrich kefir’s health qualities and organoleptic characters. Several metabolic products are generated during kefir production and account for its distinct flavour and aroma: Lactic acid, ethanol, carbon dioxide, and aroma compounds such as acetoin and acetaldehyde. During the storage process, microbiological, physicochemical, and sensory characteristics of kefir can further undergo changes, some of which improve its shelf life. Kefir exhibits many health benefits owing to its antimicrobial, anticancer, gastrointestinal tract effects, gut microbiota modulation and anti-diabetic effects. The current review presents the state of the art relating to the role of probiotics, prebiotics, additives, and different manufacturing practices in the context of kefir’s physicochemical, sensory, and chemical properties. A review of kefir’s many nutritional and health benefits, underlying chemistry and limitations for usage is presented.

## 1. Introduction

Kefir is a fermented milk drink with an acidic taste and creamy consistency produced by bacterial fermentation of kefir grains. The term kefir is derived from the word *kef*, which means ‘pleasant taste’ in Turkish. Kefir grains are the natural starter for kefir and are recovered after the fermentation process. The grains contain a mixture of microorganisms (bacteria and yeast), which coexist and interact to produce a unique fermented dairy product [1]. Kefir is prepared from raw cow, camel, goat, sheep, or buffalo milk mixed with kefir grains [2,3]. Kefir’s chemical composition depends not only on the starter-kefir grains but also on its geographical origin, the temperature, and time-related conditions of fermentation, and especially on the type and volume of the milk used [1,4]. The characteristic smell and flavour of kefir are due to the volatile and non-volatile compounds generated upon fermentation *via* lipolysis, glycolysis, and proteolysis. The physicochemical properties of kefir include an acidic pH of 4.6, alcohol of 0.5%–2%, acidic taste, and yeasty flavour. Additionally, carbon dioxide produced by the yeast flora contributes to its sharp acid and yeasty flavour [5].

Kefir is proposed as one of the factors associated with the long life expectancy of the people of Caucasus, owing to its many health benefits such as anti-stress properties, immune-modulation [6], cholesterol-lowering [7], anti-allergenic [8], anti-asthmatic, anti-microbial [9], anticancer properties [10] and chemoprevention against colon cancer [11], aside from its gastrointestinal beneficial effects [12].

Such health benefits are attributed to kefir’s protein, vitamin, lipid, mineral, amino acid, and microelement composition. Moreover, the fermentation process enriches the content of vitamins B1, B12, K, folic acid, calcium and amino acids [13], adding to kefir’s health benefits.

This review focuses on kefir’s physiochemical, sensory analysis and flavour composition in terms of how different production methods and ingredients affect the composition of kefir and ultimately affect its biological and nutritive values.

## 2. Prebiotic, Additives, and Production Methods Employed in Kefir Production

Several schemes can be employed for kefir production, all sharing the same underlying principle. Kefir is first prepared by mixing two types of milk, such as mare, goat or sheep milk [14], or by adding additives such as native inulin, to improve its beneficial effect and final texture [15]. An alternative way of producing kefir is to utilize non-dairy substrates such as fruits and molasses to produce sugary kefir, which has unique sensory properties, such as a refreshing flavour due to the presence of ethanol, a fruity aroma due to the presence of esters and a body and texture attributed to its glycerol content [16].

The traditional method of preparing dairy-based kefir used in private households is to incubate milk with kefir grains. The kefir grains are inoculated into sterilized milk and fermented at 25 °C until a pH of 4.4 is reached. The grain and milk are then separated using a sterilized plastic filter at the end of the fermentation process [17]. In contrast, water kefir is a homemade fermented beverage based on a sucrose solution with different dried and fresh fruits. In the traditional process of sugary kefir preparation, kefir grains are placed into a solution containing 8% sucrose, dried fruits (typically figs) and some slices of lemon. Fermentation for one or two days at room temperature results in a cloudy, carbonated and straw-coloured drink, poor in sugar, slightly alcoholic, and acidic [18].

**Figure 1:**
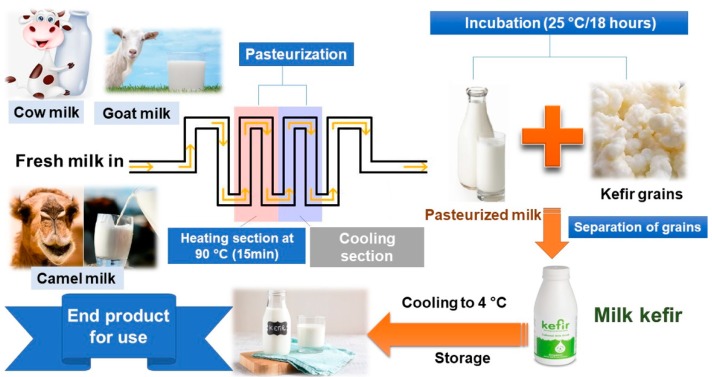
Outline diagram representing the backslopping method used for kefir production.

Backslopping is a technique used in the production of fermented food such as sourdough, idli, sauerkraut, dry sausage, beer, cheese and kefir [19]. The milk is first pasteurized at 90 °C for 15 min and is then cooled to 25 °C to improve its microbiological quality. The cooled milk is mixed with 5% kefir grains and incubated at 18–24 °C for 18 h, and the kefir grains are later separated *via* a sieve under aseptic conditions. The kefir is then stored at 4 °C (Figure 1). The fermentation step is used to speed up the microorganisms’ action and the metabolic changes occurring in milk composition [20]. A backslopping strategy is employed to increase kefir beverage generation, with a 50-fold production yield increase while maintaining the same kefir characteristics (physicochemical, microbiological and nutritional value) as traditional kefir except for the differences in the *lactobacillus kefir* population (7.94 vs. 8.89 log CFU/mL) and decreased yeast count (7.1 vs. 5.22 log CFU/mL) [17]. This method is considered cheap and reliable, especially in less developed countries, with only a few shortcomings observed in product consistency and microbiological diversity.

Kefir production faces more than one challenge, owing to the unique and diverse microflora of kefir grain, milk type, incubation time and storage conditions. The sensory, physicochemical properties and the quality of kefir products hindered the mass production of kefir on an industrial scale [21]. Such limitations may be due to the microbial diversity and interaction that influence the final product quality. Additional study is warranted to improve and standardise production at an industrial level [22].

Due to kefir’s short shelf life and high storage and packaging costs, the trend towards having dry kefir in powder form appears warranted. Both spray drying and freeze drying are used to produce powdered kefir [23]. Spray drying is the most common technique employed in the dairy powder industry due to its reduced cost, quick drying time, efficient drying and efficient moisture removal levels. However, in spray drying, some decrease in microorganism viability occurs concurrently with a loss of aroma and flavour. Factors that affect the survival of kefir bacteria after drying include spray drying inlet and outlet temperatures, atomization type, direction of airflow and initial microorganism counts [24]. Freeze drying is known as the best drying process and can maintain sensory properties and the viability of the bacteria. However, freeze drying has a high cost and longer processing time that constrict its use in the food industry [25,26]. For mass production, spray drying is accepted for its product stability; however, the main limitation is the loss of microorganism viability during the drying process [23].

Altogether, these results show that kefir production is influenced by several factors including raw materials, production technology, and storage conditions that all need to be optimized in parallel to achieve the best product quality.

## 3. Physicochemical Parameters of Kefir in the Context of Its Different Production Methods

**Figure 2:**
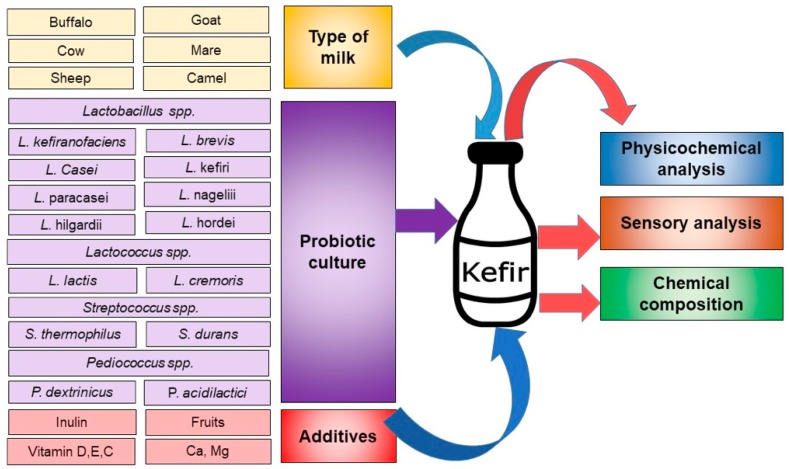
Major variables including milk, culture and additives employed in kefir production that affect quality and chemical composition.

Typical kefir consists of 90% moisture, 3.0% protein, 0.2% lipid, 6.0% sugar, 0.7% ash, 1.0% lactic acid, 0.48% alcohol and 201.7–277.0 mL/L CO_2_, all of which are dependent on the amount of kefir grain [1]. The chemical composition of kefir depends mostly on the type of milk used, the grains, or mixture of cultures, additives and the technology employed in its production. Figure 2 shows an outline of these variables [27]. The composition of dry matter, fat, protein, total carbohydrates, and ash content depends on the milk type. Cow milk kefir was found to be enriched in protein, fat, and lactose compared to that prepared using camel milk, concurrent with low dry matter and ash content [3]. The alcohol, protein, fat and ash levels were found to be influenced by kefir grain levels and pH fermentation. For example, using 1% kefir grain at a pH of 4.5 led to a decrease in alcohol level to 0.3% in goat milk kefir, compared to 1% alcohol when using 5% kefir grain at the same pH value [28]. Such a decrease in alcohol level in kefir can be favoured in certain parts of the world, e.g., Islamic countries in which alcoholic beverages are not allowed.

The starter culture employed in kefir production exhibits a significant effect on its viscosity and on chemical composition [22]. The kefir microbial community encompasses a complex mixture of lactic acid bacteria (LAB) (*Leuconostocs*, *Lactobacilli*, *Streptococci*, *lactococci*, *Enterobacter*, *Acinetobacter*, *Enterococcus*, and *Pseudomonas* spp.), acetic acid bacteria and yeasts (*Kluyveromyces*, *Candida*, *Torulopsis*, *Saccharomyces*, *Rhodotorula* and *Zygosaccharomyces*) (Table 1) [14,29]. Yeast plays a vital role in establishing an environment that allows for the growth of kefir bacteria, aside from the production of several key metabolites such as peptides, amino acids, vitamins, ethanol and CO_2_ that contribute ultimately to kefir’s flavour and aroma [30,31] and its several health benefits. In Brazil, kefir grains are used to ferment milk in private households. Brazilian kefir is characterized by the presence of three microbial populations: yeast (*Saccharomyces cerevisiae)*, lactic acid bacteria and gram-negative bacteria *(Lactobacillus paracasei)* to yield kefir with lactic acid, alcohol and acetic acid. Chemical analysis revealed that the highest concentration of lactic acid (7.30 mg/mL), followed by acetic acid (6.50 mg/mL) and malic acid (4.00 mg/mL) is observed in cow milk kefir fermentation [32]. The increase in lactic acid bacterial population was found to be correlated with an increase in lactic acid levels [33], and aside from imparting the unique taste in kefir, lactic acid inhibits microorganism growth owing to decrease in pH acting as an acidulant preservative. In contrast, yeast (*S. cerevisiae*) mediates for aroma production in kefir alongside other volatile esters such as isopentyl acetate, ethyl hexanoate, ethyl octanoate, phenethyl acetate, and ethyl decanoate [34,35]. Esters are recognized for their distinctive aroma in many herbs and appear to be responsible for the predominant aromas in kefir. Tibetan kefir is characterized by Lactobacillaceae, Streptococcaceae and Leuconostocaceae families [36]. In the manufacture of Tibetan kefir, a combination of different microorganism species from *Lactococcus lactis*, *Leuconosroc mesenteroides*, *Lactobacillus kefir*, *Lactobacillus casei*, and *Kluyveromyces marxianus* are employed and produce diacetyl, ethanol, and CO_2_ at 77.23 mg/L, 4259 mg/L, and 2.12 g/L, respectively (Table 1) [37].

**Figure 3:**
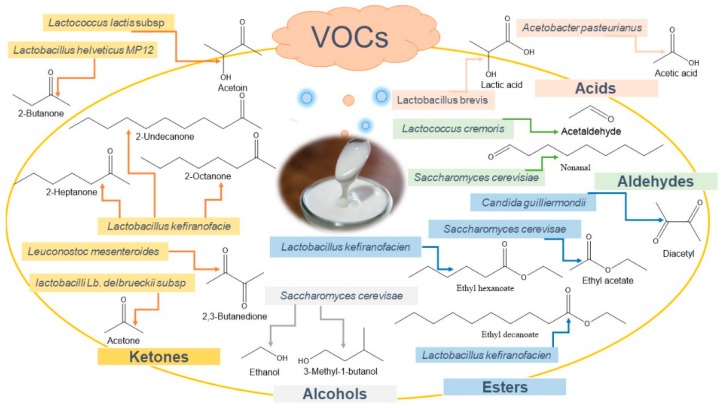
Major volatile aroma compound classes produced in kefir during fermentation.

During the fermentation of skim milk powder with kefir starter culture, volatile kefir flavour compounds were monitored using the headspace solid-phase micro-extraction (HS-SPME) method. Eight volatile flavour compounds were detected, including ethanol (39.3%), 2-butanone (31.6%), ethyl acetate (8.9%), ethyl butyrate (5.5%), acetone (3.6%), 3-hydroxy-2-butanone (acetoin, 3.3%), 2,3-butanedione (diacetyl, 2.9%) and acetaldehyde (1.7%), representative of the alcohol, ketone, ester and aldehyde classes, respectively (Figure 3). Additionally, acetone, diacetyl, ethanol, acetaldehyde and ethyl acetate increased during fermentation [38].

**Figure 4:**
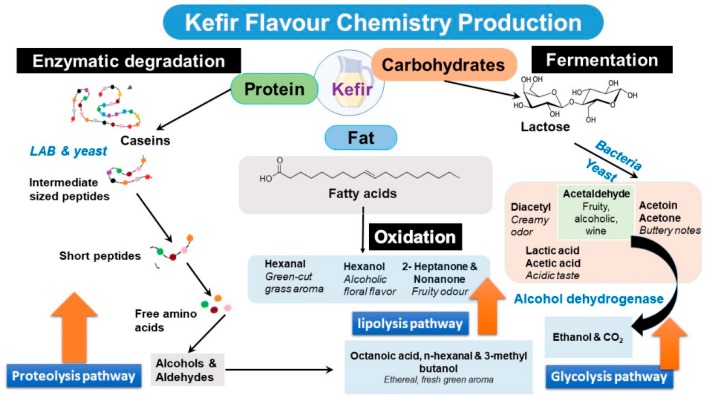
Kefir flavour chemistry production *via* proteolysis, lipolysis and glycolysis of milk macronutrients by the action of different microbes within kefir grains.

Free fatty acids (FFAs) produced *via* lipolysis in milk are responsible for the taste and aroma of several fermented milk products, including kefir (Figure 4). Indeed, fermented milk was found to contain from 5 to 10 times more FFAs than milk. For example, incubation of sheep milk inoculated with a kefir culture led to a 4.3 fold increase of its FFAs [39]. FFAs in kefir prepared using sheep milk incubated at two temperatures (23 °C and 26 °C, each for 16–18 h) showed a higher amount of FFAs at the lower temperature, concurrent with lower acetaldehyde and diacetyl levels [40]. From a sensory point of view, kefir produced at the higher temperature (26 °C) was more desirable than that produced at 23 °C. The polyunsaturated fatty acid ratio in the camel milk kefir was found to be lower than that of cow milk kefir, concurrent with a higher *Lactobacillus* ssp. count in cow milk kefir [3]. The low abundance of microbes in camel milk is attributed to the bacteriocin peptide which exhibits an antimicrobial effect that has yet to be determined.

With regard to major volatile classes contributing to kefir’s aroma, alcohol (e.g., ethanol), ketone (e.g., 3-hydroxy-2-butanone (acetoin and 2-butanone), ester (e.g., ethyl acetate) and aldehyde (e.g., acetaldehyde) were detected (Figure 3 and Figure 4). Among volatiles generated during kefir production, the content of 2-butanone was found to be stable during fermentation in contrast to ethanol. Acetoin levels depended on pH and were found to significantly decrease at pH values from 4.6 till 5 [33,38]. These findings suggest that acetoin and alcohol could provide a better readout of kefir manufacturing conditions than monitoring only 2-butanone.

Metabolomic profiling is an analytical tool that has the potential to further the identification of the components of kefir and to monitor biochemical changes due to bacterial activity and or moreover upon storage.

## 4. Sensory Analysis of the Different Kefir Types

Kefir should exhibit acceptable aroma, flavour, and good mouthfeel properties to meet consumers’ requests, all of which relate to its rheological properties. These characteristics are influenced first by the milk type used and its effect on kefir properties (textural, rheological and organoleptic properties). Kefirs produced from camel, cow, goat or ewe milk were found to have similar microbiological properties [58]. The addition of polysaccharide (0.2% xanthan) or pomegranate extract led to an increase in kefir’s stability with the best rheological and sensory properties [59,60]. The use of buffalo or cow milk with kefir grains and starter cultures was examined, with buffalo kefir found to exhibit higher viscosity and consistency with fewer modulus values compared to that made from cow milk [61]. Buffalo kefir exhibits higher yeast content resulting in a significant increase in ethanol level. The occurrence of ethanol gives the exotic, refreshing aroma of buffalo kefir [61]. Panelists judged the buffalo kefir as having improved sensory and colour properties compared to the cow kefir, suggesting that a combination of buffalo and cow milk could help improve kefir’s overall quality [61]. The addition of 2% kefir grain in goat milk improved the sensory quality; a white colour, typical kefir scent, and a non-acid taste were observed [62].

When flavour attributes in kefir, such as sour, sweet, salty, bitter, creamy, cheesy, sharp, gas, alcohol and metallic flavour tastes were compared, camel and cow milk kefirs scored differently. Camel milk kefir sample was found to be more sour, cheesy and have a sharper aroma than kefir prepared from cow milk, though its consistency and appearance had a lower score than that of cow milk kefir. Camel milk kefir received an overall better preference score from the panelists, mostly on account of its higher sourness [3].

Kefir prepared using non-animal milk, i.e., soymilk and 2% sucrose, exhibited acceptable flavour and aroma compounds. After two weeks of the cold storage, decreased levels of ethanol, acetaldehyde, diacetyl, and acetoin were noted [63]. There was no significant difference between the use of cow-soy milk mixture and cow milk in relation to pH and the acidity values or the stability of acidity during the storage period [5], while the addition of pomegranate juice and honey influenced its physicochemical, rheological and sensory properties. The addition of less than 5% pomegranate juice reduced pH value concurrent with an increase in viscosity, while the addition of more than 5% pomegranate juice substantially reduced protein and kefran concentration. Sensory analysis revealed that the addition of honey at 2.5% reduced acidity with an increase in viscosity and sweetness [64], aside from the myriad of health benefits of honey itself.

The formation of polyunsaturated fatty acids (PUFAs) in kefir made from goat and sheep milk was reported. PUFAs were found to affect the kefir aroma profile significantly. Increasing PUFA content led to the loss of the typical whey aroma (aromatics associated with whey powder) in goat kefir whereas the creamy aroma (aromatics associated with milk fat) became more prevalent in the case of sheep kefir [2].

The addition of thickening-agent additives, such as inulin, during kefir production did not significantly affect its chemical, microbial composition, odour or flavour, though it exhibited a higher viscosity value [15,61]. Thickening agents are likely to improve kefir’s overall stability and/or shelf life as in case of acidophilus milk. The addition of 1% (w/v) glucose and 10% grain for camel milk led to a reduction in its protein, fat, lactose content, viscosity, ash, dry matter, and titratable acidity compared to camel milk. Nevertheless, the higher cholesterol level (18.24 vs. 7.97 mg/100 g) in camel-based kefir might present a disadvantage compared to cow-based kefir if hyperlipidemia is a limiting factor for consumers [3]. Mare’s milk and a mixture of mare, goat and sheep milk were fermented using *mesophilic* LAB and produced kefir found to be firmer, and of higher consistency and viscosity, than mare’s milk alone [14]. The functional properties of goat kefir would present an added advantage to the human daily diet in the form of high protein, fat, total solids, vitamins and minerals [65], affirming why the inclusion of more than one type of milk is favoured for kefir production.

In summary, aroma, flavour and good mouthfeel properties appear to be influenced by additives such as kefir grain, inulin, and sucrose, whereas milk type influenced kefir’s textural and rheological properties.

## 5. Kefir’s Nutritional Value & Health Benefits

**Figure 5:**
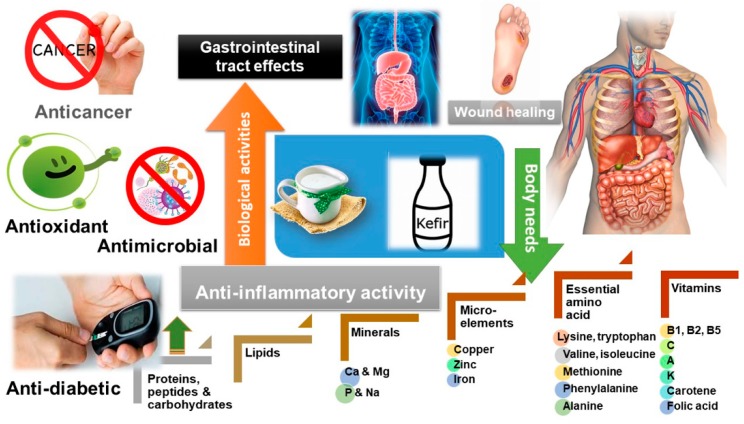
Biological properties, nutritional value, and macro- and micronutrient composition of kefir.

The number of fermented dietary supplements in the market has recently increased, owing to increased health awareness and lifestyle changes supporting (supposedly) healthy foods worldwide [33]. The nutritional values of kefir are due to its rich chemical composition, including minerals, sugars, carbohydrates, proteins, peptides, vitamins and fats (Figure 5). Aside from such chemical makeup, it is the fermentation process that further enhances kefir’s nutritional value owing to secondary bioactive ingredients such as catechin, vanillin, ferulic acid, and salicylic acid. The latter has been identified in kefir produced from peanut milk [66]. Kefir is enriched in vitamins B1, B2, B5 and C, minerals and essential amino acids that are of value for improving fitness, the healing process and homeostasis. The vitamin composition in kefir is affected by the type of milk and microbiological flora used in its production. *Propionibacterium peterssoni* and *Propionibacterium pituitosum* produced vitamin B12, whereas *Freudenreichii* subsp. *Propionibacterium Shermanii* supported more vitamin B6 production [67]. Kefir is rich in the amino acids serine, threonine, alanine, lysine, valine, isoleucine, methionine, phenylalanine and tryptophan, which play a major role in the central nervous system. Kefir also contains partially digested proteins (e.g., caseins) that aid in its digestion and absorption by the body [68]. The essential amino acids found in abundance in kefir also regulate protein, glucose and lipid metabolism and exhibit a positive effect on the regulation of body weight, maintenance of immune response and energy balance. Amino acids prevent disability and prolong the healthy life expectancy of elderly subjects [69,70], and the branched-chain amino acids that are also found in kefir improve the cognitive recovery of patients with severe traumatic brain injury [70].

Macro-elements enriched in kefir include calcium, magnesium, potassium, and sodium that aid in the utilization of carbohydrates, fats and proteins for cell growth, maintenance, and energy. Kefir also contains micro-elements, including iron, zinc, and copper, which are of special value in cellular metabolism and blood production [71].

Peptides are regarded as a unique and important class of compounds generated during milk fermentation and account for much of fermented milk products’ health benefits. In Brazil, fermented sheep’s milk provides a good source of bioactive peptides that exhibit antioxidant and antimicrobial activities [72]. The peptide F3 was purified from Tibetan kefir and exhibited antibacterial properties against *Escherichia coli* and *Staphylococcus aureus* [73]. In bovine kefir derived from *β*-casein proteolysis, 236 peptides were detected and found to display antimicrobial, antioxidant, angiotensin-converting enzyme (ACE)-inhibitory, immunomodulation and antithrombotic effects [72]. Amorim et al. identified 35 peptides in bovine milk kefir that exhibited an antihypertensive effect mediated via inhibition of ACE activity [74].

## 6. Kefir’s Biological Properties in the Context of Its Different Manufacturing Practices

The search for new bioactive chemicals is an ongoing effort. Fermented functional foods, including kefir, are a focus of this research [12]. Kefir possesses a myriad of bioactive properties to include anticancer [11], antimicrobial [73], anti-inflammatory [75], hypocholesterolemic [76], wound healing [77], antioxidant [56] and gastrointestinal aiding properties as summarized in Figure 5 [12], which will be discussed in details over the next subsections.

### 6.1. Anticancer Activity

Cancer is a major health problem worldwide, and requests for functional foods or nutritional supplements that can provide benefit in the treatment of cancer are on the rise [10].

Several studies have suggested the potential antitumor properties of kefir against breast cancer [78], colorectal cancer [79], and malignant T lymphocytes [80]. The cytotoxic effects of standard kefir extract against a panel of seven human cancer cell lines, including breast cancer (MCF-7), chronic myelogenous leukaemia (K562), lung cancer (A549), pancreatic cancer (PANC1), prostate cancer (PC3), ovarian cancer (SKOV3), and colorectal cancer (HCT116) revealed that kefir exhibits anticancer action against human K562 and HCT11 cells, with IC_50_ values of 11.36 and 17.39 mg/mL, respectively [81]. The regular consumption of kefir appears to lower colon tumour incidence in mice, suggesting that kefir mitigates against the development of pre-neoplastic lesions chemically induced by azoxymethane at 15 mL/kg for 8 weeks of consumption [82]. The use of kefir before and during doxorubicin treatment was found to prevent against doxorubicin’s heart-toxic effect in Sprague Dawley rats [83]. Usage of a mixture of 6 LAB from Japanese homemade kefir proved to be efficacious against HCT116, K562, and human natural killer KHYG-1 [84]. Donkey milk-based kefir administered at a dose of 0.5 mL/day reduced the tumour size of solid Ehrlich ascites carcinoma in a mouse model *via* its anti-proliferative action by regulating iNOS, eNOS and apoptosis induction [85]. Kefir’s cancer cell inhibition mechanism is suggested to be via cell cycle arrest and the induction of apoptosis through upregulating *BAX* and downregulating *BCL2* genes [86]. Chemicals present in kefir powder (commercial kefir in Japan) to mediate for these effects include sphingomyelin that promotes the secretion of anti-proliferative cytokines (particularly IFN-*β*) in human osteosarcoma cells [87].

The notable positive effects of kefir on cancer cell lines encourage its consumption as a functional diet in cancer prevention, especially in the case of intestinal cancer [88] and colorectal cancer [79]. Nevertheless, it should be noted that such evidence still lacks clinical trials and is mostly based on cell culture-based assays.

### 6.2. Hypocholesterolemic Effect

Hypercholesterolemia is a predisposing factor for cardiovascular disease and can lead to death [89]. Usage of kefir powder at 0.1 and 0.2% inhibited fat accumulation in adipose and liver tissues of high-fat diet (HFD)-induced obese mice. Kefir diminished bodyweight and lowered serum triacylglycerol (TG), total cholesterol (TC), and low-density lipoprotein (LDL) in mice [76]. The administration of kefir (140 mg/kg BW/day) for 4 weeks improved fatty liver status *via* decreasing TG and TC amounts in mice [90]. In HFD-C57BL/6 mice fed with orally administrated kefir (once a day for 12 weeks), a significant decrease of body weight (34.18 g vs. 40.24 g) compared to control (milk group) was observed. Kefir’s hypocholesterolemia effect is mediated *via* the up-regulation of genes involved in fatty acid oxidation [91]. Administration of the non-bacterial fraction of kefir (22 mL/kg for 4 weeks) as a treatment for HFD led to a reduction of lipid deposition in arteries [92], suggesting that chemicals, not microorganisms, mediate for such an effect. Kefir peptides significantly increase fatty acid oxidation *via* the induction of phosphorylated AMP-activated protein kinase, peroxisome proliferator-activated receptor-*α*, and hepatic carnitine palmitoyltransferase-1 in mice livers. In addition, kefir peptides significantly reduced the inflammatory response (TNF-*α*, IL-1*β* and TGF-*β*) in the liver associated with oxidative damage [93]. The insoluble peptide fraction (Fr-3) (0.1 mg/mL) present in kefir significantly decreased lipid accumulation and glycerol-3-phosphate dehydrogenase (GPDH) activity without affecting cell viability, concurrent with a reduction in mRNA expression of adipocyte-specific genes (aP2, FAS and ACC) [94]. Lactobacillus isolates from kefir grains and the administration of *Lactobacillus plantarum* Lp09 and Lp45 for 4 weeks in rats fed an enriched cholesterol diet showed significantly decreased cholesterol, TC and LDL cholesterol levels in serum as well as cholesterol and TC levels in the liver compared to the control group. Bile acid and fecal cholesterol were significantly increased after LAB consumption [95]. Hypercholesterolemic rats feeding with *Lactobacillus* *plantarum* (Lp27) from Tibetan kefir grains at a dose of 10^9^ CFU/d for 4 weeks led to reduced LDL and TC levels, without affecting HDL. The mechanism of this reduction in cholesterol absorption due to kefir consumption was mediated *via* the down-regulation of Niemann-Pick C1-Like 1 (NPC1L1 is a critical protein for intestinal cholesterol absorption) expression in Caco-2 cells [96]. Three Lactobacillus strains, including *L. plantarum* B23, *L. acidophilus* LA15 and *L.* *kefiri* D17, isolated from Tibetan kefir grains showed a reduction of TC, TG and LDL cholesterol levels in the LAB-treated rats. Additionally, both fecal cholesterol and bile acid levels were significantly increased post LAB administration [97].

The soluble non-bacterial fraction of kefir decreased lipid deposition independent of hypercholesterolemia in LDLr-/-mice [92], likely attributed to kefir peptides content found effective to improve body weight, energy intake, and protection against fatty liver [98]. Peptides derived from the microbial fermentation of milk protein account for many fermented dairy products’ reported health benefits and are likely to be same in the case of kefir. Compared to other effects, it appears that kefir’s hypocholesterolemic effect is the most elucidated at the mechanistic level, positioning it to be formulated in special functional foods used for obesity treatment.

### 6.3. Antimicrobial Activities

Kefir exerts an antibacterial effect against several microorganisms, including *S. aureus*, *E. coli*, and *Salmonella enteritidis*, higher than that achieved using standard antibiotics such as ampicillin [99]. This effect is attributed to its carbohydrate content. Consequently, kefir grain, kefir suspension, and kefiran, a water-soluble polysaccharide of the kefir grain, were tested for antimicrobial activity against several bacterial and fungal pathogens. The highest activity was revealed against *Streptococcus faecalis* KR6 and *Fusarium graminearum* CZ1. Aflatoxin is a hazardous chemical produced in stored grains that causes liver cancer, warranting its monitoring to ensure safety levels. Kefir inhibited *Aspergillus* spore formation and aflatoxin B1 production at 7–10% (*v/v*) [100]. Bovine tuberculosis is the result of infection with *Mycobacterium bovis* transmitted to humans through the consumption of infected raw and raw fermented dairy products. Kefir lowered the viability of *M. bovis* in milk mediated *via* increasing fermentation time with a directly proportional effect on *M. bovis* inhibition. A 24 h or shorter fermentation did not ensure the inactivation of *M. bovis* BCG [101]. A suspension of *Lactobacillus diolivorans* 1Z (isolated from Brazilian kefir grains) inhibited the growth of *Salmonella enterica* serovar Typhimurium with a higher survival rate (70%) than in control mice [102].

The antimicrobial peptide Bacteriocin F1, isolated from Tibetan kefir and made up of 18 amino acids, exhibited a bacteriostatic action at 62.5 μg/mL against *Escherichia. coli*. The antimicrobial activity is mediated *via* outer and inner membrane leakage of the *E. coli* cell. Bacteriocin F1 offers an added advantage of being stable against heat, pH and protease treatment, positing its usage in food preservation [73,103]. *Lactobacillus plantarum C4* isolated from kefir exerts a protective effect against intestinal infection caused by *Yersinia enterocolitica.* Such protection is further associated with a pro-inflammatory status in the intestinal mucosa and concurrent with an increase in the production of secreted immunoglobulin A (IgA) [104]. *Streptococcus mutans* and *S. sobrinus* are the main bacterial causes of dental caries. *Lactobacillus strains* (*Lactobacillus kefiranofaciens* DD2) isolated from kefir led to growth inhibition and anti-biofilm formation against both microbes [105]. *Lactobacillus* *kefiranofaciens* M1 mitigated against enterohemorrhagic *E. coli* (EHEC) infection, bacterial translocation, intestinal damage, and renal damage, whereas *L. plantarum* isolated from kefir mixture inhibited the Shigella invasion of Hep-2 cell culture [106]. Except for a few animal-based studies, most antimicrobial action for kefir was studied using direct growth inhibition assays and has yet to be generalized using in vivo based assays in live tissues post oral administration.

### 6.4. Wound Healing

A wound is a physical injury caused by the opening or breaking of the skin. Wounds are problematic for a large number of patients, with more than six million patients suffering from chronic wounds worldwide/year [107]. A topical application of a 70% kefir gel was used to cure cutaneous wounds in a Wistar rat model. Kefir enhanced wound healing compared to neomycin-clostebol after the seventh day of the experiment [108]. The efficacy was explained by the presence of bioactive ingredients, that is, the acetic acid and lactic acid produced by the bacteria. The correlation between the concentration of active substances and the efficacy of probiotic therapy was thought to be debatable, and more studies are thus warranted. In a rat model, wounds treated with *L. acidophilus* showed higher healing progress post-burn compared to Eucerin ointment. The *L. acidophilus* is believed to act *via* its anti-inflammatory action, accelerating the granulation tissue formation and re-epithelialization [109]. In diabetic foot ulcer (DFU) patients, kefir supplementation for 12 weeks led to a significant reduction in ulcer size [110]. Kefir improved proliferation and migration of human dermal fibroblast (HDF) cells, reduced IL-1*β* and transforming growth factor-*β*1 (TGF-*β*1) expression in parallel with stimulation of basic fibroblast growth factor (bFGF). Kefir’s bacteriostatic effect against *Pseudomonas aeruginosa* and *S. aureus* growth contributed to the faster healing of burn wounds [111]. The wound healing activity and antimicrobial effects of kefir gel were tested in rat burn wounds infected with *Pseudomonas aeruginosa*, using an antimicrobial kefir gel that was found to have an effect similar to the positive control, silver sulfadiazine ointment. However, the kefir gel showed a slower wound healing time [112].

### 6.5. Anti-Inflammatory and Antioxidant Activities

Ten weeks of kefir supplementation in spontaneously hypertensive rats was able to reduce inflammatory cytokine (IL-1β) expression in adipose tissue, increase anti-inflammatory cytokine (IL-10) and decrease the oxidative markers malondialdehyde (MDA) and hydroperoxides [75].

Extracellular vesicles (EV) produced from kefir can ease the TNF-induced inflammation in intestinal cells by inhibiting inflammatory cytokine production. Treatment of each kefir-derived *Lactobacillus* EV onto TNF-*α*-stimulated Caco-2 cells significantly reduced mRNA expression and secretion of IL-8. Western blot analysis revealed that such an effect was related to TNF-*α* signalling inhibition mediated by reducing p65 phosphorylation, a subunit of NF-kB. Subsequent application of kefir-derived *Lactobacillus* EV into inflammatory bowel disease-induced mice significantly alleviated associated symptoms such as body weight loss and rectal bleeding and enhanced stool consistency [113]. Kefir led to a 42% reduction of TNF-α/IL-10 and a 50% reduction of pro-inflammatory IL-6 level ratio, concurrent with an enhancement of anti-inflammatory IL-10 level [92]. The lyophilized Tibetan kefir polysaccharide extract exhibited good inhibitory action of hyaluronidase enzyme with minimal activity at 2.08 mg/mL [75]. Kefiran exhibits nitric oxide scavenging activity at 10 mg/mL comparable to that of quercetin [114].

The antioxidant capacity of kefir made from ewe milk was superior to that prepared from cow milk, posing ewes as a better source of milk to produce kefir used as an antioxidant. The biological effects of kefir from different milk types have yet to be thoroughly identified with respect to major effects. Kefir samples fermented using grain exhibited a stronger antioxidant capacity for 2,2-diphenyl-1-picrylhydrazyl (DPPH) and 2,2′-azino-di(3-ethylbenzthiazolin-sulfonate) (ABTS) than kefir samples fermented by starter culture storage. This experiment suggested that ewe milk kefir exhibited an antioxidant effect in the ABTS assay [115].

During milk fermentation, EPS isolated from Tibetan kefir grains exhibited an in vitro antioxidant activity and concentration-dependent protection of protein from oxidative damage [56]. The addition of kefir to apple juice was found to enhance its total phenolic content (TPC) and antioxidant activities [116]. Peanut milk kefir extract displayed stronger antioxidant properties than peanut milk alone, suggesting a fermentation impact of kefir grain on peanut milk’s efficacy [66]. Kefir containing a mixture of soy and cow milk exhibited higher antioxidant activity compared to cow kefir (positive control), with antioxidant action attributed to the presence of phenolic compounds that increased after fermentation [5].

Ebner et al. determined 257 peptide sequences from bovine milk kefir, of which the three peptide sequences VYPFPGPIPN, ARHPHPHLSFM and YQEPVLGPVRGPFPIIV exhibited an antioxidant effect [72]. Kefiran was found to possess a beneficial antioxidant effect against reactive oxygen species [117].

### 6.6. Anti-Diabetic Activity

Kefir has demonstrated promising effects in alleviating obesity and associated metabolic dysfunction [118]. Administration of kefir prepared using goat milk and black rice extract showed a similar effect to that of glibenclamide as an anti-diabetic agent [117]. Additionally, feeding of diabetic rats with kefir made from goat milk and soy milk demonstrated an anti-diabetic action as evidenced by a decrease in plasma glucose level concurrent with an increase in glutathione peroxidase (GPx) activity and improved insulin release [119]. An investigation of the active chemicals mediating the antidiabetic effect of kefir has yet to be conducted.

### 6.7. Gastrointestinal Tract Effects and Gut Microbiota Modulation

Kefir administered orally, once daily for four weeks at 4.3 and 10.7 g/kg BW/day, modulated gut microbiota composition and yielded anti-fatigue activity by lowering plasma lactate, ammonia and creatine kinase levels, thereby increasing physical performance, forelimb grip strength and the swimming time to exhaustion in mice [12]. Whether kefir can be provided as a supplement for athletes has yet to be fully examined. Additional evidence for kefir’s gut microbiota modulation effect was demonstrated from kefiran’s capacity to change faecal and gut microbiota of BALB/c mice via increasing the number of *Bifidobacteria* populations, without a change in *Lactobacillus* populations [120]. Pre-treatment with a microbial mixture containing bacteria and yeasts isolated from kefir inhibited *Shigella flexneri* from internalising into human intestinal epithelial cells and led to the attenuation of the inflammatory response [121]. Feeding mice with kefir reduces protozoan *Giardia intestinalis* infection and promotes the activation of different mechanisms of humoral and cellular immunity that are down-regulated by this parasitic infection [122]. Kefir supernatant and dried kefir powder both exerted radiation-protective effects on the small intestinal mucosa [123], suggesting that these kefir products can aid in alleviating radiation therapy treatment against cancer. Oral administration of kefir averted diarrhoea and enterocolitis triggered by *Clostridium difficile* [124].

In summary, many studies have demonstrated the biological activities of kefir highlighting its potential as an antioxidant, antimicrobial, anti-inflammatory, wound healing and hypocholesterolemic agent (Figure 5). Nevertheless, further studies of long-term effects on animal and human models are still needed to prove kefir’s efficacy.

## 7. Milk vs. Sugar Kefir Limitations and Safety

While kefir is well recognized for its potential health value as an excellent source of probiotics, some limitations in kefir consumption need to be recognized. These limitations are mostly due to its cholesterol-rich content [125] and potential to trigger allergic reactions. Adapting to non-dairy substrates could be an alternative way to obtain the beneficial health effects from kefir, as exemplified in sugary kefir. A brown sugar solution is the main alternative substrate used nowadays in the fermentation of kefir and produces a beverage known as sugar kefir. Sugary kefir possesses a similar microbial association as traditional milk kefir fermentation, especially lactic acid bacteria and yeast species such as *Lactobacillus*, *Leuconostoc*, *Kluyveromyces pichia* and *Saccharomyces*. Sugary kefir was found to be more effective at improving the lipid profile in mice than milk kefir [125]. As far as structure, associated microorganisms and products formed during the fermentation process are concerned, sugar kefir grains are quite similar to the milk kefir grains. Melon, carrot, onion, tomato, fennel and strawberry juices have been used as fermentable substrates to produce kefir on which LAB and yeasts could grow [16]. Among these substrates, microorganisms showed the best growth on melon juice. Esters were the major volatile compounds in onion, melon, and strawberry juices, and terpenoids were abundant in fennel and carrot. Aside from improving kefir’s aroma, onion, tomato and strawberry juices were found to contribute to its strong antioxidant effect [126]. For a review of sugary kefir’s microbiological, biochemical, and functional aspects, the work of Fiorda et al. (2017) ought to be consulted [16].

Questions of quality control and the safety of kefir material have always been relevant to the dairy industry. However, little attention has been given to the safe use of kefir. The literature contains sparse information on the safe levels of kefir intake or the amount to be consumed and the time needed to exert beneficial health effects.

Hemolysis is a common virulence factor among pathogens, with bacterial hemolytic activity being the first safety parameter evaluated in vitro. Another important safety feature is antibiotic sensitivity [127].

*Lactobacilli* are microorganisms that are useful in dairy technology (cheese, yoghurt and fermented milk) and have a long documented history of food use. *L. kefiri* strains did not cause *α*-or *β*-hemolysis and were found susceptible to tetracycline, clindamycin, streptomycin, ampicillin, erythromycin, kanamycin and gentamicin. *L. kefiri* strains could inhibit pathogens from both gram-positive and negative bacteria. The mice model receiving an oral dose of *L. kefiri* CIDCA 8348 daily (10^8^ CFU) did not show signs of pain, lethargy, dehydration or diarrhoea, or differences in food and water intake over 21 days. During necropsy, no signs of inflammation or damage were observed in any organ; no differences in the secretion of proinflammatory cytokines between treated and control mice were observed [52]. *L. mali* K8 showed pH 2.5 tolerance and resisted the damaging effects of bile salts, pepsin, and pancreatin, similar to *L. rhamnosus* (reference strain). *L. mali* K8 was found susceptible to all tested antibiotics except vancomycin. The safety of the *L. mali* K8 strain was indicated by a lack of hemolytic activity and its susceptibility to the five standard antibiotics chloramphenicol, oxacillin, tetracycline, penicillin G and ciprofloxacin [128]. The three *L. paracasei* strains (MRS55, MRS59, and M1743) showing the undesirable activity of trypsin, α-chymotrypsin, and *β*-glucuronidase were not detected [127] in 32 different LAB strains isolated from Brazilian kefir grains.

Aflatoxin G1 (AFG1) is one of the main toxic contaminants in nuts and causes potential health hazards. Hence, AFG1 reduction is one of the main concerns of food safety. Using kefir grain has a significant effect on AFG1 decontamination in pistachio nuts. The optimized biological detoxification method using 70 °C treated kefir grains could be suitable for routine removal of AFG1 [129] from pistachios. In Wistar rats, kefir supplementation with a normal dose (0.7 mL/day/animal) and high dose (3.5 mL/day/animal) for 4 weeks did not show harmful effects on animals as determined by rat growth, haematology, and blood chemistry, as well as potential pathogenicity in tissues. These findings clearly show that both the normal and high dose of kefir is safe for consumption. The results highlight that although no damage was observed in the mucosa due to the high consumption dose of kefir, the normal dose is recommended due to the most pronounced beneficial effects [130].

*Enterococcus durans* strains could inhibit various pathogens of gram-positive and negative bacteria. These strains were able to survive simulated gastrointestinal conditions and showed similar adhesion power to mucins. Notably, *E.durans* strains exhibited anti-inflammatory properties as shown by significant flagellin-induced response inhibition of Caco-2 cells. The results showed that *E. durans* does not pose a threat to the health of consumers and demonstrate its potential both as a functional food and as a source of probiotics [131].

## 8. Concluding Remarks and Future Directions in Kefir

Kefir is a popular ethnic dairy product that is constantly subjected to development; different additives of flavours, milk types, fibres, grains, and many more have been examined for the market. Kefir is made from various types of milk (cow, goat, camel, buffalo, or mare), and is usually produced by mixing two types of milk to enhance its benefits, flavor, and texture, and subjected to secondary fermentation or the addition of additives such as inulin to improve the final product properties. The fermentation of kefir grains in a raw sugar solution or sugar from fruits or vegetables without using milk is another way of producing kefir. This product is called sugary kefir. These additives and different production methods, aside from affecting palatability, also affect kefir’s physicochemical properties and health benefits. The type of milk used, the kefir grain and the fermentation conditions of production (time and temperature) should be monitored during manufacture as any changes in these variables can impact kefir’s chemical and microbiological composition.

The compiled literature in this review sheds light on some of the most noteworthy components assessed using chemical and sensory analyses. Studies reported in this review on kefir production primarily determined the influence of a single variable on the product quality or composition; the interactions between the variables have not been fully examined. It would be necessary to simultaneously investigate the influence of different variables on the quality of the final product using statistical design to optimize kefir fermentation conditions. Another recommended approach is to apply advanced data analyses to design models for comparing products, designating the most effective additives and better achievement of optimal kefir properties. Metabolomics, as a strategy for identification of detailed fermented milk composition and record of biochemical changes due to bacterial activity during the fermentation process and storage, can be readily applied to predict the sensory, nutritional, and safety measures of kefir. A myriad of macro- and micronutrients are encompassed within kefir including proteins, lipids, amino acids, and vitamins. These components account for kefir’s antibacterial, immunological, chemopreventive, and hypocholesterolemic effects and for why kefir can be consumed by people who have lactose intolerance.

Most of these health effects are based on biochemical or laboratory assays and need to be substantiated by studies in animals and humans to be more conclusive. Additionally, monitoring changes in the human gut microbiome after ingesting the different probiotics available in kefir products can provide a better understanding of its many health benefits.

## Figures and Tables

**Table 1:** List of microorganisms isolated from kefir grains, their chemical end products, and odour descriptors as typical in fermented dairy products.

Microorganisms (Kefir Type)	Produced Metabolites	Odor and FlavorDescription	Reference
*Lactobacillus kefiranofaciens* (Tibetan kefir) *Lactobacillus acidophilus* Z1L (Turkish homemade kefir) *Lactobacillus* *brevis* *Lactobacillus helveticus Z5L* *Lactobacillus casei Z7L1* (Turkish homemade kefir) *Lactobacillus harbinensis* (Water kefir) *Leuconostoc mesenteroides* *Lactococcus cremoris* *Lactococcus lactis* *Streptococcus durans* *Pediococcus dextrinicus ZN1P* *Pediococcus acidilactici ZN10P* *Pediococcus pentosaceus ZN13P* (Candida kefir) *Candida guilliermondii* *Saccharomyces Jorentinus* (Sugar kefir)	Lactic acid	Sour flavor	[41,42,43,44,45,46,47,48]
*Lactobacillus harbinensis* (Water kefir) *Leuconostoc mesenteroides* *Acetobacter pasteurianus* *Lactococcus cremoris*	Acetic acid	Vinegar, green, fruity, sour	[43]
*Lactobacillus kefiri* *Lactobacillus harbinensis* *Lactobacillus hilgardii* (Water kefir) *Leuconostoc mesenteroides* (Candida kefir) *Saccharomyces* *turicensis* *Saccharomyces florentinus* *Kluyveromyces marxianus*	Ethanol	Alcoholic flavor	[35,43,44,45,48,49,50,51,52]
*LactobacillusBrevis* *Lactobacillus kefiri* *Leuconostocmesenteroides* (Candida kefir) *Saccharomycesturicensis* *Saccharomycescerevisae* *Pichia Kurdriavzevii*	CO_2_	Sharp odor and a sour taste	[45,51,52,53]
*Lactobacillus kefiranofaciens*	Octanoic acid	Cheesy, rancid, pungent, sweet, soapy, goaty	[54]
*Lactobacillus kefiranofaciens* (Tibetan kefir) *Lactobacillus kefiri* (Brazilian kefir)	Kefiran	-	[41]
*Lactobacillus kefiranofaciens*	n-Decanoic acid	Soapy, waxy, stale, buttery, fruity, grassy, cheesy, milky	[54]
*Lactobacillus kefiranofaciens*	Ethyl decanoate	Fruity, grape, cognac	[54]
*Lactobacillus kefiranofaciens*	Ethyl hexanoate	Animal, cardboard	[54]
*Lactobacillus nagelii*, *Lactobacillus hilgardii &* *Lactobacillus hordei* (Water kefir) *Lactobacillus plantarum* YW11 (Tibet Kefir) *Lactobacillus acidophilus Z1L* (Turkish homemade kefir) *Lactobacillus helveticus Z5L* *Lactobacillus* *casei Z7Ll* *Lactococcus* *cremoris Z11S &* *Lactococcus* *lactis Z3S* (Turkish homemade kefirs) *Pediococcus dextrinicus ZN1P* *Pediococcus acidilactici ZN10P* *Pediococcus pentosaceus ZN13P* *Kazachstania unispora*	Exopolysaccharides (EPS)		[18,42,55,56]
*Lactobacillus kefiranofaciens DN1*	New exopolysaccharide (EPS), EPS_DN1	-	[57]
*Lactobacillus acidophilus Z1L &* *Lactococcuscremoris Z11S* (Turkish homemade kefir) *Pediococcus dextrinicus ZN1P* *Pediococcus* *acidilactici ZN10P* *Pediococcus* *pentosaceus ZN13P* *Candida* *guilliermondii* *Streptococcus thermophiles*	H_2_O_2_	-	[42,46,47]
*Lactobacillus hilgardii* *Saccharomyces florentinus* (Sugar kefir)	Pyruvate	-	[48,50]
*Lactobacillus hilgardii* *Saccharomyces florentinus* (Sugar kefir)	Propionate	-	[48,50]
*Lactobacillus hilgardii* *Saccharomyces florentinus* (Sugar kefir)	Acetate	-	[48,50]
*Lactobacillus hilgardii* *Saccharomyces florentinus* (Sugar kefir)	Succinate	-	[48,50]
*Lactobacillus hilgardii* *Saccharomyces florentinus* (Sugar kefir)	Fumarate	-	[48,50]
*Lactobacillus hilgardii* (Sugar kefir)	Mannitol	-	[48,50]
*Leuconostoc* *mesenteroides*	2,3-Butanedione	Buttery flavors	[43,46,47]
*Lactococcus cremoris Streptococcus thermophilus Streptococcus durans* *Candida* *guilliermondii*	Acetaldehyde	Fruity, alcoholic, wine	[47]
*Candida* *guilliermondii*	Diacetyl	Creamy odor	[18,35,41,50]
*Saccharomycescerevisae* (Brazilian kefir, water kefir, and Tibetan kefir grains)	Glycerol	-	[18,35,41,50]
*Saccharomyces* *cerevisae*	Nonanal	Green, citrus, fatty, floral	[54]
*Saccharomyces* *cerevisae*	Phenylethyl alcohol	Unclean, rose, violet-like, honey, floral, spicy	[54]
*Saccharomyces* *cerevisae*	Octanal	Green, fatty, soapy, fruity, orange peel	[54]
*Saccharomyces* *cerevisae*	Ethyl acetate	Solvent, pineapple, fruity, apples	[54]
*Saccharomyces* *cerevisae*	3-Methyl-butanol	Penetrating, alcohol, wine-like, plastic	[54]

(-) Represents unreported.
